# Increased risk of cancer and cancer-related mortality in middle-aged Korean women with prediabetes and diabetes: a population-based study

**DOI:** 10.4178/epih.e2023080

**Published:** 2023-08-28

**Authors:** Thi Xuan Mai Tran, Soyeoun Kim, Huiyeon Song, Boyoung Park

**Affiliations:** 1 Department of Preventive Medicine, Hanyang University College of Medicine, Seoul, Korea; 2 Institute for Health and Society, Hanyang University, Seoul, Korea; 3 Department of Epidemiology and Biostatistics, Graduate School of Public Health, Hanyang University, Seoul, Korea; 4 Institute of Bioscience and Biotechnology, Hanyang University, Seoul, Korea

**Keywords:** Diabetes mellitus, Prediabetic state, Neoplasms, Mortality, Women

## Abstract

**OBJECTIVES:**

This study investigated the risk of developing and dying from all types of cancer, as well as cancer-specific mortality, in women diagnosed with prediabetes and diabetes.

**METHODS:**

We included women aged ≥40 years who underwent cancer screening from 2009 to 2014 with follow-up until 2020. Diabetes status was determined based on fasting plasma glucose levels, self-reported history of diabetes, and the use of antidiabetic medication. We quantified the risk of cancer and mortality in the prediabetes and diabetes groups, relative to the normoglycemia group, by calculating adjusted hazard ratios (aHRs).

**RESULTS:**

The study included 8,309,393 participants with a mean age of 52.7±9.7 years. Among these participants, 522,894 cases of cancer and 193,283 deaths were detected. An increased risk of cancer was observed in both the prediabetes group (aHR, 1.03; 95% confidence interval [CI], 1.02 to 1.04) and the diabetes group (aHR, 1.13; 95% CI, 1.12 to 1.14). The highest risk was identified in those with diabetes who developed liver (aHR, 1.72; 95% CI, 1.66 to 1.79), pancreatic (aHR, 1.68; 95% CI, 1.60 to 1.76), and gallbladder cancer (aHR, 1.43; 95% CI, 1.36 to 1.51). Women with prediabetes and diabetes exhibited a 1.07-fold (95% CI, 1.05 to 1.08) and 1.38-fold (95% CI, 1.36 to 1.41) increased risk of death from cancer, respectively.

**CONCLUSIONS:**

Both prediabetes and diabetes were associated with an elevated risk of cancer, as well as an increased risk of death from cancer, in middle-aged Korean women. However, the degree of risk varied depending on the specific site of the cancer.

## GRAPHICAL ABSTRACT

[Fig f2-epih-45-e2023080]

## INTRODUCTION

Type 2 diabetes mellitus (hereafter referred to as diabetes) and cancer are chronic diseases that place a significant burden on the healthcare system. Both have been linked epidemiologically and biologically. There is considerable evidence suggesting that individuals with diabetes are at an increased risk of developing cancer [1,2]. One potential explanation for this correlation between these conditions is the commonality of certain risk factors, such as aging, obesity, smoking, an unhealthy diet, and physical inactivity [3]. Furthermore, potential biological mechanisms that may link diabetes and cancer include hyperinsulinemia, hyperglycemia, and inflammation [3]. A recent study using Mendelian randomization found a causal effect of diabetes on certain cancer types [4]. In addition to the increased risk of cancer, studies have shown that diabetes and prediabetes also increase the risk of death from all causes and cancer [5,6].

Although the literature consistently points to an increased risk of cancer and mortality in individuals with diabetes and prediabetes, several unresolved issues remain. Firstly, there is a possibility that some of the existing evidence may be biased due to reverse causality, where cancer itself triggers the onset of diabetes [7]. Secondly, there is a growing recognition that even non-diabetic levels of hyperglycemia, as seen in impaired fasting glucose, could be linked to an increased risk of cancer and cancer-related mortality [6,8]. However, few studies have investigated both the risk of cancer and cancer-related mortality in relation to diabetes and prediabetes. Thirdly, the risk of gynecologic cancers, such as cervical and ovarian cancer, has been less explored, and the findings for breast cancer, intrahepatic cholangiocarcinoma, colorectal cancer, and endometrial cancer have been inconsistent and inconclusive [2]. Therefore, there is a need for well-executed and well-designed prospective observational studies to evaluate the risk of cancer in individuals with diabetes and prediabetes [3]. Additionally, while the prevalence of diabetes and prediabetes is higher in Asians than in Westerners with the same body mass index (BMI) [9], most of the findings regarding the risk of death in diabetes and prediabetes are primarily derived from Western populations [5], and evidence in Asian populations is limited.

To address the above-mentioned limitations, we conducted a large-scale cohort study involving over 8 million women who had undergone cancer screening. We evaluated both the general risk of developing cancer and the specific risk associated with prediabetes and diabetes in women. Regarding mortality outcomes, we examined the risk of death from any cause as well as from cancerrelated causes.

## MATERIALS AND METHODS

### Study settings and study population

We utilized data from the National Health Insurance Service’s (NHIS) National Health Information Database (NHID). In Korea, the NHIS, a government-managed single insurer, covers approximately 97% of the population, while the Medical Aid program covers the remaining 3%. The NHIS offers health screening examinations to eligible individuals to evaluate risk factors and detect chronic diseases such as cardiovascular diseases, diabetes, and cancer [10]. This study used data from the National Breast Cancer Screening Program, which is embedded in the NHIS database.

Our initial database included women aged ≥ 40 years who participated in both a biennial breast cancer screening program and a general health screening between 2009 and 2014. The general health screening included a fasting plasma glucose (FPG) measurement. Participants were tracked until either a cancer diagnosis, death, or December 31, 2020, whichever occurred first. For women who underwent more than one screening during the study period, we used the information gathered during their first screening. We excluded participants who did not have an FPG measurement, as well as those aged under 40 or over 75 at the time of screening. We also excluded participants with a history of any type of cancer. To avoid the potential inclusion of prevalent cancer cases at screening, we further excluded those who were diagnosed with any type of cancer or who died within 90 days following their screening. The final dataset included 8,309,393 women (Supplementary Material 1).

### Assessment of the exposure

To define the study exposure, we used FPG measurements taken during health screenings, as well as information about antidiabetic medication use gathered from self-administered questionnaires. Glucose levels were measured after a minimum fasting period of 8 hours. During the health examination, participants were asked to disclose whether they had ever received a diabetes diagnosis and if they were currently using antidiabetic medication.

Following the American Diabetes Association criteria [11], we defined prediabetes as an FPG level of 100-125 mg/dL without a previous diabetes diagnosis and without taking antidiabetic medication, and diabetes as an FPG level ≥ 126 mg/dL, or a history of diabetes diagnosis and current use of antidiabetic medication. The normoglycemia group was defined as patients with FPG < 100 mg/dL with no diabetes diagnosis and not taking antidiabetic medication.

### Assessment of cancer development outcomes

The primary outcome of interest in this study was the incidence of invasive cancer. The NHIS-NHID database includes data from all inpatient and outpatient clinic visits that have been assigned a disease code according to the International Classification of Diseases, 10th revision (ICD-10). The ICD-10 codes C00-C97 are specifically assigned to malignant neoplasms. To enhance the accuracy of cancer case identification, we utilized both ICD-10 codes (C00-C97) and the catastrophic illness code designated for cancer patients in Korea [12]. The analysis incorporated the following outcomes: all cancer sites and selected specific cancer sites. The selected cancer sites included the following malignancies: malignant neoplasms of the lip, oral cavity, and pharynx (C10-C14), esophagus (C15), stomach (C16), colon (C18 and C19), rectum (C20), liver (C22), gallbladder (C23 and C24), pancreas (C25), larynx (C32), lung (C33 and C34), breast (C50), cervix (C53), uterus (C54), ovary (C56), kidney (C64), bladder (C67), brain (C70 and C72), and thyroid (C73).

### Assessment of mortality outcomes

Mortality data, including the date and cause of death, were obtained from nationwide death certificate data provided by the Korea National Statistical Office. Our analysis included the following mortality outcomes: death from any cause and cancer-related causes. Deaths from specific common cancer sites were also assessed, including the stomach (C16), colorectal (C18-C20), liver (C22), gallbladder (C23 and C24), pancreatic (C25), lung (C33 and C34), breast (C50), cervix (C53), and ovary (C56).

### Assessment of covariates

Height and weight were measured by trained medical staff during the health examination, and BMI was calculated as weight in kilograms divided by the square of height in meters. Information on current health behaviors and other health-related factors was collected using self-administered standardized questionnaires during the health screening. Regarding comorbidities, information on the history of hypertension, dyslipidemia, ischemic heart disease, and stroke was collected using a self-reported questionnaire, and by asking the participants whether they had been diagnosed with the abovementioned diseases by a physician. We included age at screening, BMI, age at menarche, menopausal status, age at menopause, family history of cancer, parity, breastfeeding, oral contraceptive use, smoking status, drinking status, physical activity, comorbidity status (hypertension, dyslipidemia, ischemic heart disease, and stroke), and hormone replacement therapy use as covariates.

### Statistical analysis

Descriptive statistics were used to characterize the study participants in each group (normoglycemia, prediabetes, and diabetes). Categorical variables are reported as frequencies (percentages), and continuous variables are expressed as means and standard deviations. The incidence rate of cancer and the mortality rate were calculated by dividing the number of events within the follow-up period by the total person-years and presented as the incidence per 100,000 person-years.

To quantify the association between diabetes, prediabetes status, and the risk of cancer, we performed Cox proportional hazards regression analysis to estimate hazard ratios (HRs) and 95% confidence intervals (CIs). In the regression models, the normoglycemia group was used as the reference group and 2 regression models were used for diabetes and prediabetes. For the association between diabetes, prediabetes status, and cancer mortality risk, competing risk analysis was used, considering deaths from other causes as competing events. The proportional hazard assumption was tested using Kaplan–Meier curves and parallel lines of the survival distribution function. The first model was adjusted for age at screening. The second regression model was adjusted for age at screening, BMI, age at menarche, menopausal status, age at menopause, family history of cancer, breastfeeding, oral contraceptive use, smoking status, drinking status, physical activity, hormone replacement therapy uses, and comorbidity status (hypertension, dyslipidemia, ischemic heart disease, and stroke). Missing covariate values were treated as dummy variables (separate categories).

For cancer incidence outcomes, we conducted a stratified analysis of the association between diabetes, prediabetes, and cancer risk by age group (40-49, 50-59, and ≥ 60 years), menopausal status (premenopausal and postmenopausal), and BMI status (< 23, 23 to < 25, and ≥ 25 kg/m2). The results of the subgroup analysis for cancer risk are presented in the Supplementary Materials 2-6. Statistical analyses were performed using SAS version 9.4 (SAS Institute Inc., Cary, NC, USA), and a 2-sided p-value < 0.05 was considered statistically significant.

### Ethics statement

The Institutional Review Board of Hanyang University College of Medicine (approval No. HYUIRB-202106-003-1) approved this study, and the National Health Insurance Sharing Service system approved the use of the NHIS database.

## RESULTS

Of the 8,309,393 women included in the analysis, 5,796,928 (69.8%) were included in the normoglycemia group, 1,825,545 (22.0%) in the prediabetes group, and 686,920 (8.3%) in the diabetes group (Table 1, Supplementary Material 1). The mean age of the total cohort was 52.7± 9.7 years. The mean age of the diabetes group (54.2± 9.7 years) and diabetes group (59.3± 9.3 years) was higher than that of the normoglycemia group (51.4± 9.4 years).

During a median follow-up of 10 years (interquartile range, 8.1-11.0), 522,894 incident cancer cases were identified (Table 2). The incidence rate per 100,000 person-years was 636.0 (95% CI, 633.9 to 638.1) in the normoglycemia group, 705.3 (95% CI, 701.3 to 709.3) in the prediabetes group, and 878.4 (95% CI, 871.1 to 885.6) in the diabetes group. In all 3 groups, thyroid cancer was the most common incident cancer, followed by breast cancer. Despite the higher risk of almost all types of cancer in the prediabetes and diabetes groups than in the normoglycemia group, the incidence of the 2 most common types of cancers (breast and thyroid cancers) was highest in normoglycemic women, followed by those in the prediabetes and diabetes groups.

**Figure 1.**
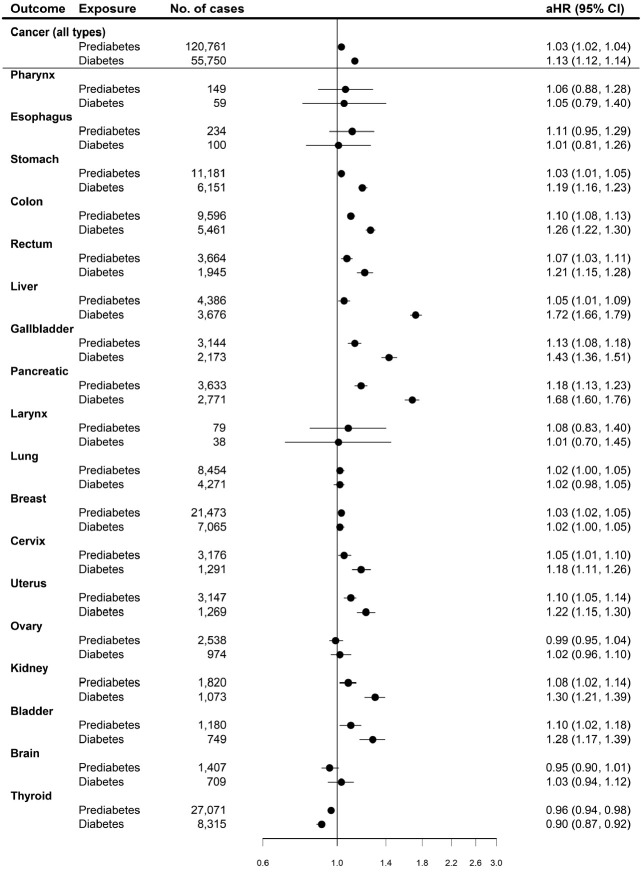
Associations between prediabetes, diabetes, and subsequent risk of cancer. aHR, adjusted hazard ratio; CI, confidence interval.

Prediabetes and diabetes status were significantly associated with elevated cancer risk before and after adjustment for other covariates (Figure 1, Supplementary Material 2). The adjusted HRs for cancer risk compared with normoglycemia were 1.03 (95% CI, 1.02 to 1.04) in the prediabetes group and 1.13 (95% CI, 1.12 to 1.14) in the diabetes group. The association varied by cancer site, and a significant association between diabetes and cancer risk was observed in the following cancer sites: stomach, colon, rectum, liver, gallbladder, pancreatic, larynx, cervix, uterus, kidney, and bladder. Among the analyzed cancer sites, the highest risk of diabetes was observed in liver cancer, with an adjusted HR of 1.72 (95% CI, 1.66 to 1.79), followed by pancreatic and bladder cancer (HR of pancreatic cancer: 1.68; 95% CI, 1.36 to 1.51; HR of bladder cancer: 1.43; 95% CI, 1.36 to 1.51), relative to the normoglycemia group. The prediabetes group had significant risk of developing the following cancer sites: stomach (HR, 1.03; 95% CI, 1.01 to 1.05), colon (HR, 1.10; 95% CI, 1.08 to 1.13), rectum (HR, 1.07; 95% CI, 1.03 to 1.11), liver (HR, 1.05; 95% CI, 1.01 to 1.09), gallbladder (HR, 1.13; 95% CI, 1.08 to 1.18), pancreas (HR, 1.18; 95% CI, 1.13 to 1.23), cervix (HR, 1.05; 95% CI, 1.01 to 1.10), uterus (HR, 1.10; 95% CI, 1.05 to 1.14), kidney (HR, 1.08; 95% CI, 1.02 to 1.14), and bladder (HR, 1.10; 95% CI, 1.02 to 1.18). In the stratified analysis of cancer development outcomes, a similar pattern of association was observed compared to that in the main analysis (Supplementary Materials 3-5).

In total, 193,283 deaths were recorded in our cohort during the follow-up period (Supplementary Material 6). The all-cause mortality rate was 188.8 (95% CI, 187.6 to 190.0) per 100,000 personyears in the normoglycemia group, 262.0 (95% CI, 259.6 to 264.4) in the prediabetes group, and 718.3 (95% CI, 711.7 to 724.9) in the diabetes group. The mortality rate due to cancer (per 100,000 person-years) was 76.5 (95% CI, 75.8 to 77.3) in the normoglycemia group, 103.0 (95% CI, 101.5 to 104.5) in the prediabetes group, and 197.5 (95% CI, 194.0 to 200.9) in the diabetes group. In all 3 groups, lung cancer was the most common cause of cancer, followed by pancreatic and liver cancer.

The all-cause mortality rate in the prediabetes and diabetes groups was 1.08-fold (95% CI, 1.07 to 1.09) and 1.88-fold (95% CI, 1.85 to 1.90) higher than that in women with normoglycemia, respectively (Table 3). Compared to women with normoglycemia, women with prediabetes and diabetes had 1.06-fold (95% CI, 1.05 to 1.08) and 1.36-fold (95% CI, 1.34 to 1.39) increased risks of death due to cancer, respectively. Prediabetes was significantly associated with an elevated risk of death from stomach (HR, 1.10; 95% CI, 1.03 to 1.18), colorectal (HR, 1.07; 95% CI, 1.02 to 1.14), gallbladder (HR, 1.11; 95% CI, 1.05 to 1.18), pancreatic (HR, 1.16; 95% CI, 1.10 to 1.21), and breast (HR, 1.12; 95% CI, 1.04 to 1.22) cancers. In the diabetes group, the risk of death from all assessed cancer sites was significantly increased, with the highest risk observed for liver cancer (HR, 1.75; 95% CI, 1.65 to 1.85) and pancreatic cancer (HR, 1.62; 95% CI, 1.53 to 1.71).

## DISCUSSION

Our analysis, conducted on a cohort of over 8 million women, revealed that both diabetes and prediabetes were significantly associated with an increased risk of subsequent cancer, all-cause mortality, and cancer-related death. A systematic review demonstrated that individuals with diabetes had a 1.25-fold (95% CI, 1.19 to 1.31) increased risk of death from cancer compared to those without diabetes [13], which is relatively similar to the HR of 1.38 found in our analysis. The risks varied depending on the site of the cancer. Compared to the normoglycemic group, the HRs for diabetes were notably high for liver and pancreatic cancer, in terms of both incidence and mortality. Coughlin et al. [14] discovered a significant association between diabetes and fatal colon, pancreatic, bladder, liver, and breast cancers in a cohort of United States women. These significant cancer sites align with our results, albeit with a slightly higher risk.

We found a positive association between diabetes and an elevated risk of cancer incidence and mortality in digestive organs, including the stomach, colon, rectum, liver, gallbladder, and pancreas. A positive association between diabetes/prediabetes and the risk of pancreatic cancer and pancreatic cancer-specific mortality was found in the current study, previous studies [4,15,16], and previous meta-analyses [2]. The findings from another cohort study conducted on the Korean population suggested that hyperglycemia, insulin resistance, and hyperinsulinemia were independent predictors of pancreatic cancer mortality, not only in individuals with diabetes but also in those without diabetes [16]. Additionally, a Mendelian randomization study utilizing genome-wide data revealed a correlation between genetically elevated fasting insulin levels and an increased risk of pancreatic cancer [15]. However, no evidence was found regarding an elevated risk of pancreatic cancer associated with type 2 diabetes or dyslipidemia [15].

Glucose metabolism disorders, such as diabetes and prediabetes, have been linked to an increased risk of colon and rectal cancers [17,18], as found in the current study. Several meta-analyses also found a significant relationship between diabetes and colorectal cancer risk and colorectal cancer-specific mortality [19]. In this study, we found that compared to women with normal glucose levels, those with prediabetes showed a slight but significant increase in gastric cancer risk, while those with diabetes exhibited an even higher risk. This contradicts the results of a Swedish cohort study, which found no significant link between prediabetes or diabetes and gastric adenocarcinoma [20]. However, meta-analyses suggest that the association between prediabetes, diabetes, and gastric cancer may be more pronounced in women, particularly those of Asian descent [21]. The proposed mechanisms connecting diabetes and gastric cancer include shared risk factors such as obesity and insulin resistance, as well as *Helicobacter pylori* infection, salt intake, hyperglycemia, and certain medications like insulin and metformin [21]. Compared to other types of cancer, the risk of liver and gallbladder cancers associated with diabetes and prediabetes has been more extensively documented in previous epidemiological studies [22], which is consistent with our results. The significant increase in hepatic cancer risk among individuals with diabetes and prediabetes may be attributed to the disruption of liver homeostasis caused by hyperglycemia, insulin resistance, and hyperinsulinemia [23].

In this study, diabetes was not significantly associated with the overall risk of breast cancer. However, a subgroup analysis revealed an increased risk of breast cancer in women aged 60 years and older, as well as in postmenopausal women with impaired glucose levels and diabetes. The relationship between the risk of breast cancer and diabetes, however, remains unclear. A pooled analysis of 20 studies indicated a significantly increased risk of breast cancer in women with diabetes, yet the pooled relative risk from 5 cohort studies showed no association [24]. Recent evidence further suggests no link between diabetes and breast cancer, potentially due to the protective effect of metformin, a medication used to treat diabetes, which may help reduce the incidence of estrogen-positive breast cancer [25]. However, our analysis did not take into account the type of antidiabetic medication used or the subtypes of breast cancer, due to the lack of available relevant data.

Our findings support an increased risk of uterine and cervical cancer in groups with prediabetes and diabetes. However, no correlation was discovered with the risk of ovarian cancer. This is consistent with previous studies that have identified an increased risk of cervical cancer in patients with diabetes [4,26]. Diabetes has been associated with poor survival in cervical and ovarian cancer patients [27,28], and researchers have suggested diabetes as a prognostic marker of these cancers. While we cannot directly compare the survival rates of cancer patients with the mortality rates of the general population, our results do reveal higher mortality rates from cervical and ovarian cancer in women with diabetes compared to those with normal blood sugar levels. This could potentially corroborate the findings in cancer patients.

Diabetes was found to be associated with an increased risk of cancers related to the urinary tract, including bladder and kidney cancers. This finding is consistent with a previous meta-analysis that reported a positive association between diabetes and kidney cancer risk [29]. However, a recent large-scale cohort study found a significant association between diabetes and renal cell carcinoma risk exclusively in women (aHR, 1.53; 95% CI, 1.14 to 2.04), but not in men [30]. The observation of an increased risk of kidney cancer only in women with diabetes might be due to the interaction between diabetes with other hormone-related exposures, such as oral contraceptives or postmenopausal hormone replacement [31]. This hypothesis necessitates further investigation.

Limited and inconsistent evidence has been reported regarding the association between diabetes and the risk of lung cancer, particularly in women. These inconsistencies may be due to factors such as small sample sizes, variations in exposure ascertainment, or the exclusion or adjustment of significant covariates like BMI or smoking habits [32]. In this large study adjusted for important covariates, we found no association between diabetes or prediabetes and the risk of lung cancer, as reported in recent studies [32,33]. However, diabetes was associated with an increased risk of death from lung cancer in women, which is consistent with previous findings [34]. Several mechanisms have been proposed to explain this poor outcome, including increased tumor metastasis and progression via fibroblast growth factor 2, changes in endothelial cell function, and alterations to the baseline membrane caused by hyperglycemia [35].

Our findings suggest a reduced risk of thyroid cancer in women with diabetes and prediabetes compared to women with normal blood sugar levels. Previous studies exploring the link between diabetes and thyroid cancer have yielded inconsistent results, with some suggesting an increased risk [36] or no relationship [37]. However, a case-control study conducted within a Korean population demonstrated a lower risk of thyroid cancer in individuals with diabetes, particularly in women [38]. Most prior research on the relationship between diabetes and thyroid cancer has also taken into account diabetes treatment. Therefore, future studies should incorporate factors such as glucose levels, diabetes treatment, and the duration of diabetes when assessing the risk of thyroid cancer.

The prevalence of prediabetes increased dramatically to 27% in Korea in 2018 [39], presenting new challenges for public health interventions related to diabetes management [40]. Previous studies have also reported an association between prediabetes and an increased risk of death from cancer [6]. Our research further identified an elevated risk of cancer incidence and mortality in women with prediabetes, particularly in relation to nearly all digestive tract cancers. Therefore, individuals with prediabetes should be closely monitored as they constitute a high-risk group for both cancer incidence and mortality.

The strengths of this study lie in its large female population and the accurate, population-based assessment of all new cancer cases and deaths. Additionally, by excluding cases that developed before and within 90 days of the baseline cancer screening, we were able to evaluate the natural progression from diabetes to cancer development and cancer-related deaths, aspects not considered in previous studies on cancer mortality.

However, our study has several limitations. First, our database did not include postprandial glucose levels, preventing us from defining prediabetes using this measure. We also lacked information on the duration of diabetes and the type of antidiabetic medication used. While studies have suggested a protective effect of metformin, an antidiabetic medication, against cancer, our scant information prevented us from assessing the impact of metformin or other antidiabetic medications on cancer risk. Second, although we adjusted our analysis for hypertension and dyslipidemia, we did not have specific information on the types of medication used to treat these conditions. As a result, we were unable to assess the impact of these medications on the main association. Third, our findings were derived from a cohort of individuals undergoing cancer screening, which could introduce selection bias. Therefore, caution should be exercised when generalizing our findings. Furthermore, our analysis did not take into account other important risk factors for individual cancer sites, such as *H. pylori* infection in gastric cancer or hepatitis B virus infection in liver cancer.

In conclusion, we found that prediabetes and diabetes were independently associated with an overall increased risk of cancer, with stronger associations for malignancies in the liver and pancreas in women. Additionally, both prediabetes and diabetes were associated with an elevated risk of death from cancer. These results underscore the importance of prevention and management strategies to mitigate the burden of cancer and premature cancer-related deaths, not only in individuals diagnosed with diabetes but also in those with prediabetes.

## DATA AVAILABILITY

This study used NHIS-NHID data (research number: NHIS-2019-1-064). Data are available through the Korean National Health Insurance Sharing Service (NHISS).

## Figures and Tables

**Figure f2-epih-45-e2023080:**
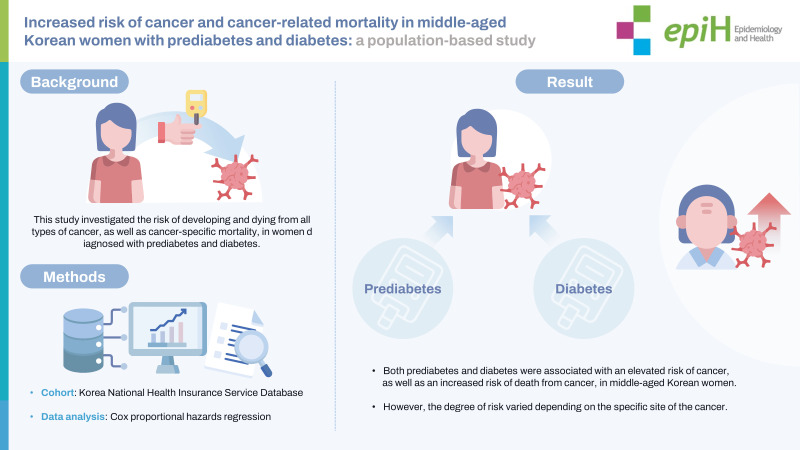


**Table 1.** Characteristics of participants in the cohort according to diabetes status

Characteristics	Diabetes status		
	Normoglycemia (n=5,796,928)	Prediabetes (n=1,825,545)	Diabetes (n=686,920)
Age at study entry (yr)	51.4±9.4	54.2±9.7	59.3±9.3
BMI (kg/m^2^)	23.3±3.3	24.5±3.4	25.4±3.6
Fasting glucose (mg/dL)	88.4±7.1	107.6±6.6	145.4±47.8
Age (yr)			
40-49	2,706,404 (46.7)	625,853 (34.3)	109,544 (16.0)
50-59	1,817,968 (31.4)	629,364 (34.5)	210,307 (30.6)
≥60	1,272,556 (22.0)	570,328 (31.2)	367,069 (53.4)
Obesity status			
No (BMI <25 kg/m^2^)	2,849,777 (49.2)	639,508 (35.0)	172,723 (25.1)
Yes (BMI ≥25 kg/m^2^)	2,946,155 (50.8)	1,185,791 (65.0)	514,055 (74.8)
Missing	996 (0.0)	246 (0.0)	142 (0.0)
Menopausal status			
Premenopausal	3,159,981 (54.5)	796,803 (43.7)	183,814 (26.8)
Postmenopausal	2,561,870 (44.2)	1,006,171 (55.1)	494,837 (72.0)
Missing	75,077 (1.3)	22,571 (1.2)	8,269 (1.2)
Age at menopause (yr)			
<52	1,513,380 (26.1)	585,593 (32.1)	289,252 (42.1)
≥52	879,402 (15.2)	363,409 (19.9)	184,627 (26.9)
Premenopausal/missing	3,404,146 (58.7)	876,543 (48.0)	213,041 (31.0)
Family history of cancer			
No	4,342,457 (74.9)	1,390,001 (76.1)	539,025 (78.5)
Yes	1,454,471 (25.1)	435,544 (23.9)	147,895 (21.5)
Age at menarche (yr)			
<15	1,733,490 (29.9)	450,326 (24.7)	128,563 (18.7)
≥15	3,913,947 (67.5)	1,329,402 (72.8)	540,782 (78.7)
Missing	149,491 (2.6)	45,817 (2.5)	17,575 (2.6)
Parity			
Nulliparous	382,062 (6.6)	105,482 (5.8)	34,002 (5.0)
1 child or more	5,414,866 (93.4)	1,720,063 (94.2)	652,918 (95.1)
Breastfeeding			
Never	1,227,334 (21.2)	312,512 (17.1)	73,499 (10.7)
Ever	4,569,594 (78.8)	1,513,033 (82.9)	613,421 (89.3)
Oral contraceptive use			
Never	4,741,219 (81.8)	1,474,225 (80.8)	540,789 (78.7)
Ever	1,055,709 (18.2)	351,320 (19.2)	146,131 (21.3)
Physical activity			
No	1,614,924 (27.9)	539,915 (29.6)	208,851 (30.4)
Yes	4,182,004 (72.1)	1,285,630 (70.4)	478,069 (69.6)
Missing			
Smoking status			
Never smoked	5,470,431 (94.4)	1,716,693 (94.0)	645,058 (93.9)
Ever smoked	326,497 (5.6)	108,852 (6.0)	41,862 (6.1)
Drinking frequency during the last year			
No	4,473,848 (77.2)	1,394,086 (76.4)	594,115 (86.5)
More than 1 day/wk	1,323,080 (22.8)	431,459 (23.6)	92,805 (13.5)
Hormone replacement therapy after menopause			
Never use	2,116,860 (36.5)	856,042 (46.9)	434,026 (63.2)
Ever use	520,087 (9.0)	172,700 (9.5)	69,080 (10.1)
Premenopausal	3,159,981 (54.5)	796,803 (43.7)	183,814 (26.8)
Comorbidity status			
Stroke (yes)	37,517 (0.7)	16,393 (0.9)	15,857 (2.3)
Ischemic heart disease (yes)	92,513 (1.6)	42,806 (2.3)	39,888 (5.8)
Hypertension (yes)	784,476 (13.5)	427,281 (23.4)	328,051 (47.8)
Dyslipidemia (yes)	168,516 (2.9)	82,820 (4.5)	72,470 (10.6)

Values are presented as mean±standard deviation or number (%). BMI, body mass index.

**Table 2.** Incidence rate^1^ of cancer in women with normoglycemia, prediabetes, and diabetes according to cancer site

Outcomes (ICD-10)	Total no. of cases	Normal		Glucose impairment		Diabetes	
		No. of cases	Incidence rate (95% CI)	No. of cases	Incidence rate (95% CI)	No. of cases	Incidence rate (95% CI)
Cancer (all types)	522,894	346,383	636.0 (633.9, 638.1)	120,761	705.3 (701.3, 709.3)	55,750	878.4 (871.1, 885.6)
Specific cancer site							
Pharynx (C10-14)	635	427	0.8 (0.7, 0.9)	149	0.9 (0.7, 1.0)	59	0.9 (0.7, 1.2)
Esophagus (C15)	904	570	1.0 (1.0, 1.1)	234	1.4 (1.2, 1.5)	100	1.6 (1.3, 1.9)
Stomach (C16)	46,995	29,663	54.5 (53.8, 55.1)	11,181	65.3 (64.1, 66.5)	6,151	96.9 (94.5, 99.3)
Colon (C18, C19)	38,098	23,041	42.3 (41.8, 42.9)	9,596	56.0 (54.9, 57.2)	5,461	86 (83.8, 88.3)
Rectum (C20)	15,000	9,391	17.2 (16.9, 17.6)	3,664	21.4 (20.7, 22.1)	1,945	30.6 (29.3, 32.0)
Liver (C22)	18,782	10,720	19.7 (19.3, 20.1)	4,386	25.6 (24.9, 26.4)	3,676	57.9 (56.0, 59.8)
Gallbladder (C23, C24)	12,172	6,855	12.6 (12.3, 12.9)	3,144	18.4 (17.7, 19.0)	2,173	34.2 (32.8, 35.7)
Pancreatic (C25)	14,186	7,782	14.3 (14.0, 14.6)	3,633	21.2 (20.5, 21.9)	2,771	43.7 (42.0, 45.3)
Larynx (C32)	310	193	0.4 (0.3, 0.4)	79	0.5 (0.4, 0.6)	38	0.6 (0.4, 0.8)
Lung (C33, C34)	34,757	22,032	40.5 (39.9, 41.0)	8,454	49.4 (48.3, 50.4)	4,271	67.3 (65.3, 69.3)
Breast (C50)	98,490	69,952	128.4 (127.5, 129.4)	21,473	125.4 (123.7, 127.1)	7,065	111.3 (108.7, 113.9)
Cervix (C53)	13,932	9,465	17.4 (17.0, 17.7)	3,176	18.5 (17.9, 19.2)	1,291	20.3 (19.2, 21.4)
Uterus (C54)	13,410	8,994	16.5 (16.2, 16.9)	3,147	18.4 (17.7, 19.0)	1,269	20 (18.9, 21.1)
Ovary (C56)	11,679	8,167	15.0 (14.7, 15.3)	2,538	14.8 (14.2, 15.4)	974	15.3 (14.4, 16.3)
Kidney (C64)	7,442	4,549	8.4 (8.1, 8.6)	1,820	10.6 (10.1, 11.1)	1,073	16.9 (15.9, 17.9)
Bladder (C67)	4,663	2,734	5.0 (4.8, 5.2)	1,180	6.9 (6.5, 7.3)	749	11.8 (11.0, 12.6)
Brain (C70, C71, C72)	6,258	4,142	7.6 (7.4, 7.8)	1,407	8.2 (7.8, 8.6)	709	11.2 (10.3, 12.0)
Thyroid (C73)	123,557	88,171	161.9 (160.8, 163.0)	27,071	158.1 (156.2, 160.0)	8,315	131.0 (128.2, 133.8)

ICD-10, International Classification of Diseases, 10th revision; No., number of deaths; CI, confidence interval.

1 Incidence rate per 100,000 person-years.

**Table 3.** Risk of mortality in participants with prediabetes and diabetes mellitus^1^

Outcomes	Hazard ratios relative to the normoglycemia group			
	Prediabetes		Diabetes	
	Model 1	Model 2	Model 1	Model 2
Death from any cause	1.08 (1.06, 1.09)	1.08 (1.07, 1.09)	1.93 (1.91, 1.95)	1.88 (1.85, 1.90)
Death from cancer	1.07 (1.05, 1.09)	1.06 (1.05, 1.08)	1.38 (1.35, 1.41)	1.36 (1.34, 1.39)
Specific cancer site				
Stomach	1.07 (1.01, 1.15)	1.10 (1.03, 1.18)	1.27 (1.17, 1.37)	1.34 (1.24, 1.45)
Colorectal	1.08 (1.02, 1.14)	1.07 (1.02, 1.14)	1.39 (1.30, 1.48)	1.40 (1.31, 1.49)
Liver	1.05 (0.99, 1.11)	1.03 (0.98, 1.09)	1.75 (1.66, 1.86)	1.75 (1.65, 1.85)
Gallbladder	1.13 (1.06, 1.20)	1.11 (1.05, 1.18)	1.49 (1.40, 1.60)	1.47 (1.38, 1.58)
Pancreatic	1.18 (1.12, 1.24)	1.16 (1.10, 1.21)	1.68 (1.59, 1.78)	1.62 (1.53, 1.71)
Lung	1.04 (1.00, 1.09)	1.04 (1.00, 1.09)	1.14 (1.09, 1.20)	1.13 (1.07, 1.19)
Breast	1.14 (1.05, 1.23)	1.12 (1.04, 1.22)	1.42 (1.28, 1.58)	1.38 (1.24, 1.54)
Cervix	1.08 (0.95, 1.22)	1.07 (0.95, 1.22)	1.29 (1.10, 1.52)	1.33 (1.13, 1.57)
Ovary	1.05 (0.96 ,1.14)	1.05 (0.97, 1.15)	1.12 (1.00, 1.25)	1.13 (1.01, 1.27)

Values are presented as hazard ratio (95% confidence interval).

1 Model 1 was adjusted for age at screening; Model 2 was adjusted for age at screening, body mass index, age at menarche, menopausal status, age at menopause, family history of cancer, parity, breastfeeding, oral contraceptive use, smoking status, drinking status, physical activity, comorbidity status, and hormone replacement therapy use.

## SUPPLEMENTARY MATERIALS

Supplementary Material 1. Flow Diagram of Selection of Eligible Population

Supplementary Material 2. Risk of cancer in participants with prediabetes and diabetes by cancer site and the age-adjusted Cox regression model

Supplementary Material 3. Risk of cancer in participants with prediabetes and diabetes according to cancer site and age group

Supplementary Material 4. Risk of cancer in participants with prediabetes and diabetes according to cancer site and menopausal status

Supplementary Material 5. Risk of cancer in participants with prediabetes and diabetes according to the cancer site and body mass index

Supplementary Material 6. All-cause and cancer-specific mortality rates in women with normoglycemia, prediabetes, and diabetes
