# Supplementary material for: Increased risk of cancer and cancer-related mortality in middle-aged Korean women with prediabetes and diabetes: a population-based study
Source: Epidemiol Health. 2023 Aug 28;45:e2023080. doi: 10.4178/epih.e2023080 (PMC10867518; doi:10.4178/epih.e2023080)
Supplement: Supplementary Material 1. — Flow Diagram of Selection of Eligible Population [file epih-45-e2023080-Supplementary-1.docx]

**Supplementary Material**

Supplementary Material 1. Flow Diagram of Selection of Eligible Population

Supplementary Material 2. Risk of cancer in participants with prediabetes and diabetes by cancer site and the age-adjusted Cox regression model

Supplementary Material 3. Risk of cancer in participants with prediabetes and diabetes according to cancer site and age group

Supplementary Material 4. Risk of cancer in participants with prediabetes and diabetes according to cancer site and menopausal status

Supplementary Material 5. Risk of cancer in participants with prediabetes and diabetes according to the cancer site and body mass index

Supplementary Material 6. All-cause and cancer-specific mortality rates in women with normoglycemia, prediabetes, and diabetes

**
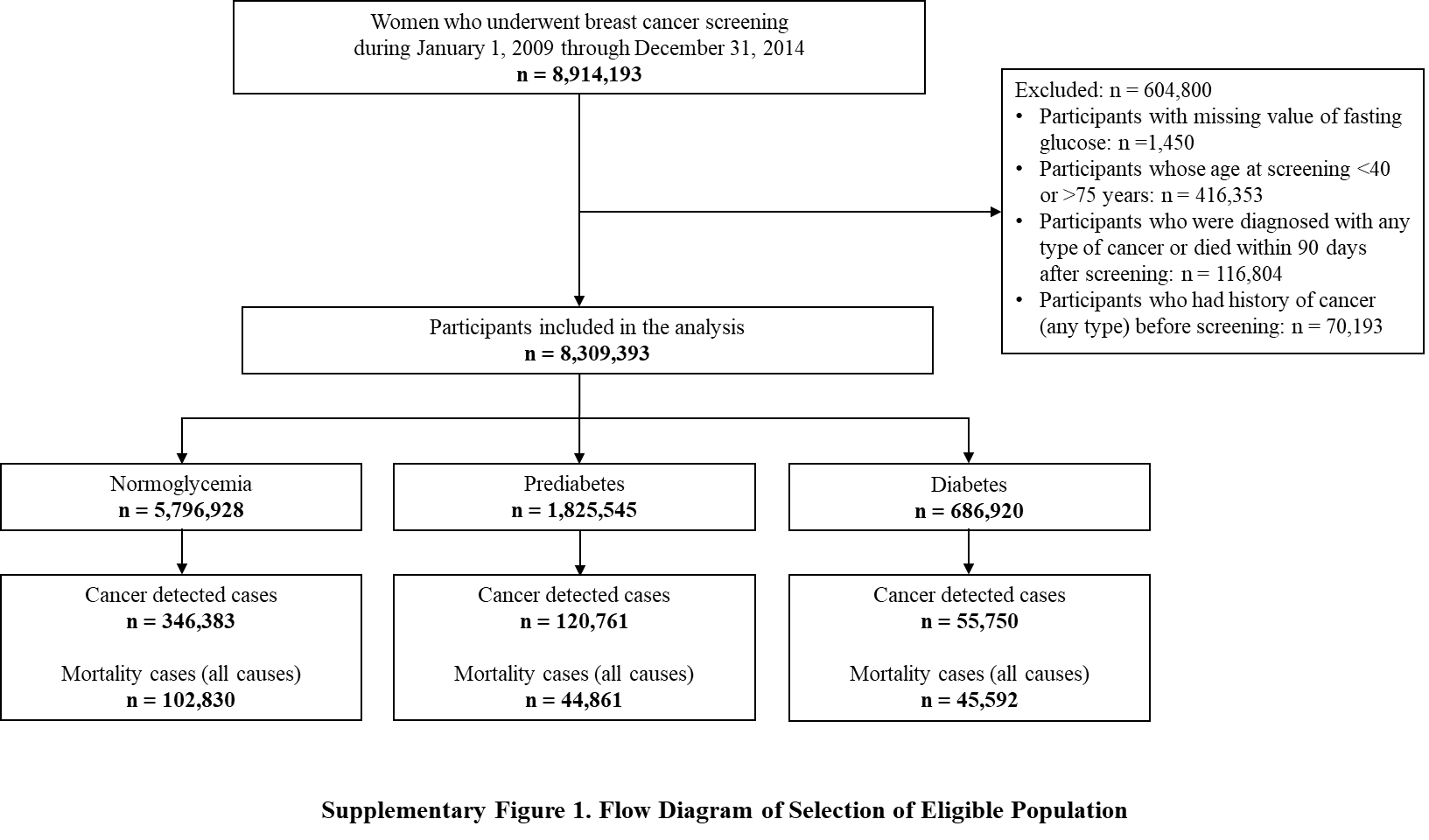
**
