# Supplementary material for: Increased risk of cancer and cancer-related mortality in middle-aged Korean women with prediabetes and diabetes: a population-based study
Source: Epidemiol Health. 2023 Aug 28;45:e2023080. doi: 10.4178/epih.e2023080 (PMC10867518; doi:10.4178/epih.e2023080)
Supplement: Supplementary Material 2. — Risk of cancer in participants with prediabetes and diabetes by cancer site and the age-adjusted Cox regression model [file epih-45-e2023080-Supplementary-2.docx]

**Supplementary Material 2. Risk of cancer in participants with prediabetes and diabetes by cancer site and the age-adjusted Cox regression model**

| **Outcome** | **Hazard ratios relative to the normal group** | | | |
| --- | --- | --- | --- | --- |
|  | **Prediabetes** | | **Diabetes** | |
|  | **HR** | **(95% CI)** | **HR** | **(95% CI)** |
| Cancer (all types) | **1.04** | **(1.04–1.05)** | **1.16** | **(1.15–1.18)** |
| Specific cancer site |  |  |  |  |
| Pharynx (C10–C14) | 1.04 | (0.86–1.26) | 0.99 | (0.75–1.31) |
| Esophagus (C15) | 1.11 | (0.95–1.29) | 0.96 | (0.78–1.20) |
| Stomach (C16) | **1.03** | **(1.01–1.05)** | **1.17** | **(1.14–1.21)** |
| Colon (C18, C19) | **1.12** | **(1.09–1.15)** | **1.29** | **(1.25–1.33)** |
| Rectum (C20) | **1.08** | **(1.04–1.13)** | **1.22** | **(1.16–1.29)** |
| Liver (C22) | **1.07** | **(1.04–1.11)** | **1.75** | **(1.68–1.82)** |
| Gallbladder (C23, C24) | **1.16** | **(1.11–1.21)** | **1.47** | **(1.39–1.54)** |
| Pancreatic (C25) | **1.21** | **(1.16–1.25)** | **1.75** | **(1.67–1.83)** |
| Larynx (C32) | 1.09 | (0.84–1.42) | 1.05 | (0.74–1.50) |
| Lung (C33, C34) | 1.02 | (1.00–1.05) | 1.03 | (1.00–1.06) |
| Breast (C50) | **1.04** | **(1.03–1.06)** | **1.06** | **(1.03–1.09)** |
| Cervix (C53) | **1.06** | **(1.02–1.11)** | **1.15** | **(1.09–1.22)** |
| Uterus (C54) | **1.15** | **(1.11–1.20)** | **1.34** | **(1.26–1.42)** |
| Ovary (C56) | 0.99 | (0.95–1.03) | 1.03 | (0.96–1.10) |
| Kidney (C64) | **1.16** | **(1.09–1.22)** | **1.55** | **(1.45–1.66)** |
| Bladder (C67) | **1.11** | **(1.04–1.19)** | **1.33** | **(1.23–1.45)** |
| Brain (C70–C72) | 0.97 | (0.91–1.03) | 1.07 | (0.99–1.17) |
| Thyroid (C73) | **1.04** | **(1.02–1.05)** | 1.01 | (0.99–1.02) |

Abbreviations: HR: Hazard ratio, CI: Confidence interval.

The model was adjusted for age at screening.

Bold values indicate statistically significant results.
