# Supplementary material for: Increased risk of cancer and cancer-related mortality in middle-aged Korean women with prediabetes and diabetes: a population-based study
Source: Epidemiol Health. 2023 Aug 28;45:e2023080. doi: 10.4178/epih.e2023080 (PMC10867518; doi:10.4178/epih.e2023080)
Supplement: Supplementary Material 3. — Risk of cancer in participants with prediabetes and diabetes according to cancer site and age group [file epih-45-e2023080-Supplementary-3.docx]

**Supplementary Material 3. Risk of cancer in participants with prediabetes and diabetes according to cancer site and age group**

| **Outcome** | **Hazard ratios relative to normoglycemia group** | | | |
| --- | --- | --- | --- | --- |
|  | **Prediabetes** | | **Diabetes** | |
|  | **No. of cases** | **aHR (95% CI)** | **No. of cases** | **aHR (95% CI)** |
| **Age group 40 to 49 years** |  |  |  |  |
| Cancer (all types) | 31,473 | **1.03 (1.01–1.04)** | 5,851 | **1.05 (1.02–1.08)** |
| Specific cancer site |  |  |  |  |
| Pharynx (C10–C14) | 39 | 1.10 (0.77–1.57) | 7 | *NA* |
| Esophagus (C15) | 24 | *NA* | 8 | *NA* |
| Stomach (C16) | 1,712 | 1.01 (0.96–1.07) | 383 | **1.26 (1.14–1.40)** |
| Colon (C18, C19) | 1,329 | **1.11 (1.04–1.18)** | 273 | **1.21 (1.06–1.37)** |
| Rectum (C20) | 660 | **1.10 (1.01–1.20)** | 139 | **1.25 (1.05–1.48)** |
| Liver (C22) | 430 | 1.01 (0.91–1.13) | 161 | **1.94 (1.64–2.29)** |
| Gallbladder (C23, C24) | 222 | 1.16 (1.00–1.35) | 62 | **1.58 (1.21–2.07)** |
| Pancreatic (C25) | 294 | 1.13 (0.99–1.28) | 108 | **2.07 (1.68–2.54)** |
| Larynx (C32) | 30 | 1.57 (0.79–3.10) | 12 | *NA* |
| Lung (C33, C34) | 880 | 0.99 (0.92–1.06) | 161 | 0.99 (0.84–1.16) |
| Breast (C50) | 8,888 | 1.02 (0.99–1.04) | 1,377 | 0.93 (0.88–0.99) |
| Cervix (C53) | 1,099 | **1.09 (1.02–1.16)** | 204 | **1.20 (1.04–1.39)** |
| Uterus (C54) | 1,079 | 1.07 (1.00–1.14) | 274 | **1.35 (1.19–1.53)** |
| Ovary (C56) | 793 | 0.91 (0.84–0.98) | 170 | 1.09 (0.94–1.28) |
| Kidney (C64) | 381 | **1.22 (1.08–1.37)** | 102 | **1.48 (1.20–1.82)** |
| Bladder (C67) | 82 | 0.88 (0.70–1.12) | 20 | *NA* |
| Brain (C70–C72) | 289 | 1.02 (0.89–1.16) | 55 | 1.03 (0.78–1.36) |
| Thyroid (C73) | 10,968 | 1.02 (1.00–1.04) | 1,867 | **0.93 (0.89–0.97)** |
| **Age group 50 to 59** |  |  |  |  |
| Cancer (all types) | 40,077 | **1.03 (1.02–1.04)** | 14,824 | **1.12 (1.10–1.13)** |
| Specific cancer site |  |  |  |  |
| Pharynx (C10–C14) | 42 | 0.77 (0.55–1.08) | 19 | 1.10 (0.68–1.79) |
| Esophagus (C15) | 80 | 1.15 (0.88–1.49) | 21 | *NA* |
| Stomach (C16) | 3,253 | **1.06 (1.01–1.10)** | 1,316 | **1.27 (1.20–1.35)** |
| Colon (C18, C19) | 2,775 | **1.13 (1.08–1.18)** | 1,157 | **1.38 (1.30–1.47)** |
| Rectum (C20) | 1,134 | **1.08 (1.01–1.16)** | 466 | **1.31 (1.18–1.45)** |
| Liver (C22) | 1,193 | 0.98 (0.92–1.05) | 707 | **1.66 (1.53–1.81)** |
| Gallbladder (C23, C24) | 745 | **1.13 (1.04–1.24)** | 393 | **1.66 (1.48–1.86)** |
| Pancreatic (C25) | 1,013 | **1.23 (1.14–1.32)** | 485 | **1.64 (1.48–1.81)** |
| Larynx (C32) | 25 | *NA* | 15 | *NA* |
| Lung (C33, C34) | 2,560 | 0.98 (0.93–1.02) | 880 | 0.96 (0.90–1.03) |
| Breast (C50) | 7,754 | **1.06 (1.03–1.08)** | 2,519 | 1.04 (1.00–1.09) |
| Cervix (C53) | 1,026 | 1.02 (0.95–1.09) | 428 | **1.31 (1.18–1.45)** |
| Uterus (C54) | 1,331 | **1.07 (1.01–1.14)** | 487 | **1.15 (1.04–1.27)** |
| Ovary (C56) | 931 | 1.05 (0.97–1.13) | 306 | 1.06 (0.94–1.20) |
| Kidney (C64) | 639 | 1.00 (0.91–1.10) | 298 | **1.20 (1.05–1.36)** |
| Bladder (C67) | 324 | **1.17 (1.02–1.33)** | 147 | **1.44 (1.20–1.73)** |
| Brain (C70-C72) | 434 | 0.90 (0.81–1.01) | 172 | 1.02 (0.87–1.20) |
| Thyroid (C73) | 10,645 | 0.98 (0.96 – 1.00) | 3,338 | **0.90 (0.87–0.93)** |
| **Age group ≥ 60 years** |  |  |  |  |
| Cancer (all types) | 49,211 | **1.04 (1.03–1.05)** | 35,075 | **1.16 (1.14–1.17)** |
| Specific cancer site |  |  |  |  |
| Pharynx (C10–C14) | 68 | 1.34 (0.99–1.81) | 33 | 1.05 (0.71–1.56) |
| Esophagus (C15) | 130 | 1.12 (0.91–1.38) | 71 | 0.98 (0.75–1.28) |
| Stomach (C16) | 6,216 | 1.02 (0.99–1.05) | 4,452 | **1.15 (1.12–1.20)** |
| Colon (C18, C19) | 5,492 | **1.08 (1.05–1.12)** | 4,031 | **1.23 (1.18–1.27)** |
| Rectum (C20) | 1,870 | 1.05 (0.99–1.11) | 1,340 | **1.17 (1.10–1.25)** |
| Liver (C22) | 2,763 | **1.08 (1.03–1.13)** | 2,808 | **1.72 (1.64–1.80)** |
| Gallbladder (C23, C24) | 2,177 | **1.11 (1.06–1.17)** | 1,718 | **1.37 (1.29–1.45)** |
| Pancreatic (C25) | 2,326 | **1.15 (1.09–1.21)** | 2,178 | **1.65 (1.57–1.74)** |
| Larynx (C32) | 42 | 0.99 (0.69–1.43) | 23 | *NA* |
| Lung (C33, C34) | 5,014 | **1.05 (1.01–1.08)** | 3,230 | 1.04 (1.00–1.08) |
| Breast (C50) | 4,831 | **1.07 (1.04–1.11)** | 3,169 | **1.13 (1.08–1.17)** |
| Cervix (C53) | 1,051 | 1.07 (0.99–1.15) | 659 | 1.10 (1.00–1.20) |
| Uterus (C54) | 737 | **1.15 (1.05–1.26)** | 508 | **1.31 (1.18–1.45)** |
| Ovary (C56) | 814 | 1.02 (0.94–1.11) | 498 | 1.00 (0.91–1.11) |
| Kidney (C64) | 800 | 1.05 (0.97–1.15) | 673 | **1.31 (1.19–1.44)** |
| Bladder (C67) | 774 | 1.09 (1.00–1.19) | 582 | **1.25 (1.13–1.37)** |
| Brain (C70-C72) | 684 | 0.95 (0.87–1.04) | 482 | 1.02 (0.92–1.14) |
| Thyroid (C73) | 5,458 | 0.98 (0.95–1.01) | 3,110 | **0.89 (0.86–0.93)** |

Abbreviations: NA: Not available due to the small number of cases, HR: Hazard ratio, CI: Confidence interval

The model was adjusted for age at screening, body mass index, age at menarche, menopausal status, age at menopause, family history of cancer, parity, breastfeeding, oral contraceptive use, smoking status, drinking status, physical activity, comorbidity status, and hormone replacement therapy use.

Bold values indicate statistically significant results.
