# Supplementary material for: Increased risk of cancer and cancer-related mortality in middle-aged Korean women with prediabetes and diabetes: a population-based study
Source: Epidemiol Health. 2023 Aug 28;45:e2023080. doi: 10.4178/epih.e2023080 (PMC10867518; doi:10.4178/epih.e2023080)
Supplement: Supplementary Material 5. — Risk of cancer in participants with prediabetes and diabetes according to the cancer site and body mass index [file epih-45-e2023080-Supplementary-5.docx]

**Supplementary Material 5. Risk of cancer in participants with prediabetes and diabetes according to the cancer site and body mass index**

| **Outcome** | **Hazard ratios relative to the normoglycemia group** | | | |
| --- | --- | --- | --- | --- |
|  | **Prediabetes** | | **Diabetes** | |
|  | **No. of cases** | **aHR (95% CI)** | **No. of cases** | **aHR (95% CI)** |
| **BMI < 23** |  |  |  |  |
| Cancer (all types) | 37,638 | **1.03 (1.01–1.04)** | 12,780 | **1.12 (1.1–1.14)** |
| Specific cancer site |  |  |  |  |
| Pharynx (C10–C14) | 48 | 0.86 (0.63–1.17) | 24 | *NA* |
| Esophagus (C15) | 106 | 1.13 (0.91–1.41) | 35 | 1.04 (0.73–1.49) |
| Stomach (C16) | 3,404 | 1.02 (0.98–1.06) | 1,488 | **1.20 (1.14–1.27)** |
| Colon (C18, C19) | 2,775 | **1.10 (1.05–1.15)** | 1,223 | **1.25 (1.17–1.33)** |
| Rectum (C20) | 1,135 | **1.09 (1.02–1.17)** | 448 | **1.18 (1.07–1.31)** |
| Liver (C22) | 1,197 | 1.04 (0.98–1.11) | 780 | **1.66 (1.53–1.80)** |
| Gallbladder (C23, C24) | 803 | **1.13 (1.04–1.22)** | 512 | **1.56 (1.41–1.72)** |
| Pancreatic (C25) | 984 | **1.17 (1.08–1.25)** | 705 | **1.90 (1.75–2.08)** |
| Larynx (C32) | 29 | *NA* | 14 | *NA* |
| Lung (C33, C34) | 2,856 | 1.04 (1.00–1.08) | 1,160 | 1.03 (0.96–1.09) |
| Breast (C50) | 7,320 | **1.03 (1.01–1.06)** | 1,515 | 1.00 (0.95–1.06) |
| Cervix (C53) | 1,099 | 1.04 (0.98–1.11) | 304 | 1.10 (0.98–1.24) |
| Uterus (C54) | 789 | 0.99 (0.91–1.07) | 199 | 0.98 (0.84–1.13) |
| Ovary (C56) | 853 | 0.97 (0.90–1.04) | 234 | 0.99 (0.87–1.14) |
| Kidney (C64) | 433 | 1.00 (0.90–1.12) | 220 | **1.34 (1.16–1.56)** |
| Bladder (C67) | 335 | 1.05 (0.93–1.19) | 174 | **1.24 (1.05–1.46)** |
| Brain (C70–C72) | 430 | 0.95 (0.85–1.06) | 159 | 0.97 (0.82–1.15) |
| Thyroid (C73) | 8,625 | 1.02 (1.00–1.04) | 1,782 | **0.88 (0.84–0.93)** |
| **23 ≤ BMI < 25** |  |  |  |  |
| Cancer (all types) | 30,061 | **1.03 (1.01–1.04)** | 12,617 | **1.10 (1.08–1.12)** |
| Specific cancer site |  |  |  |  |
| Pharynx (C10–C14) | 41 | 1.14 (0.79–1.64) | 13 | *NA* |
| Esophagus (C15) | 50 | 1.21 (0.87–1.70) | 21 | *NA* |
| Stomach (C16) | 2,847 | 1.03 (0.99–1.08) | 1,475 | **1.20 (1.13–1.27)** |
| Colon (C18, C19) | 2,405 | **1.11 (1.06–1.17)** | 1,194 | **1.18 (1.10–1.25)** |
| Rectum (C20) | 952 | **1.11 (1.03–1.20)** | 453 | **1.22 (1.10–1.35)** |
| Liver (C22) | 1,062 | 1.07 (1.00–1.15) | 788 | **1.67 (1.54–1.81)** |
| Gallbladder (C23, C24) | 750 | 1.09 (1.00–1.19) | 499 | **1.45 (1.31–1.61)** |
| Pancreatic (C25) | 959 | 1.22 (1.13–1.31) | 670 | **1.68 (1.54–1.84)** |
| Larynx (C32) | 17 | *NA* | 9 | *NA* |
| Lung (C33, C34) | 2,214 | 1.05 (1.00–1.10) | 980 | 0.95 (0.89–1.02) |
| Breast (C50) | 5,191 | **1.04 (1.01–1.07)** | 1,522 | 1.00 (0.95–1.06) |
| Cervix (C53) | 767 | 1.05 (0.97–1.14) | 301 | **1.23 (1.08–1.39)** |
| Uterus (C54) | 741 | 1.09 (1.00–1.18) | 253 | **1.16 (1.01–1.33)** |
| Ovary (C56) | 645 | 1.01 (0.93–1.11) | 216 | 0.96 (0.83–1.12) |
| Kidney (C64) | 483 | **1.18 (1.06–1.32)** | 232 | **1.29 (1.11–1.49)** |
| Bladder (C67) | 295 | 1.06 (0.92–1.21) | 171 | 1.19 (1.00–1.41) |
| Brain (C70–C72) | 345 | 0.94 (0.83–1.06) | 174 | 1.11 (0.94–1.31) |
| Thyroid (C73) | 6,782 | 0.98 (0.95–1.01) | 1,821 | **0.83 (0.79–0.87)** |
| **BMI ≥ 25** |  |  |  |  |
| Cancer (all types) | 53,062 | **1.03 (1.02–1.04)** | 30,353 | **1.14 (1.12–1.15)** |
| Specific cancer site |  |  |  |  |
| Pharynx (C10–C14) | 60 | 1.28 (0.92–1.76) | 22 | *NA* |
| Esophagus (C15) | 78 | 1.04 (0.78–1.37) | 44 | 0.95 (0.67–1.34) |
| Stomach (C16) | 4,930 | 1.03 (1.00–1.07) | 3,188 | **1.18 (1.13–1.23)** |
| Colon (C18, C19) | 4,416 | **1.09 (1.05–1.13)** | 3,044 | **1.29 (1.24–1.35)** |
| Rectum (C20) | 1,577 | 1.02 (0.96–1.08) | 1,044 | **1.21 (1.13–1.30)** |
| Liver (C22) | 2,127 | 1.02 (0.97–1.08) | 2,108 | **1.73 (1.64–1.83)** |
| Gallbladder (C23, C24) | 1,591 | **1.13 (1.06–1.20)** | 1,162 | **1.35 (1.26–1.45)** |
| Pancreatic (C25) | 1,690 | **1.15 (1.08–1.22)** | 1,396 | **1.55 (1.45–1.66)** |
| Larynx (C32) | 33 | 1.08 (0.70–1.65) | 15 | *NA* |
| Lung (C33, C34) | 3,384 | 1.00 (0.96–1.04) | 2,131 | 1.05 (1.00–1.10) |
| Breast (C50) | 8,962 | **1.05 (1.02–1.08)** | 4,028 | **1.05 (1.02–1.09)** |
| Cervix (C53) | 1,310 | 1.07 (1.00–1.14) | 686 | **1.20 (1.10–1.31)** |
| Uterus (C54) | 1,617 | **1.13 (1.06–1.20)** | 817 | **1.28 (1.18–1.38)** |
| Ovary (C56) | 1,040 | 1.00 (0.93–1.08) | 524 | 1.08 (0.98–1.19) |
| Kidney (C64) | 904 | 1.04 (0.96–1.13) | 621 | **1.25 (1.14–1.38)** |
| Bladder (C67) | 550 | **1.17 (1.05–1.30)** | 404 | **1.36 (1.20–1.53)** |
| Brain (C70–C72) | 632 | 0.95 (0.87–1.05) | 376 | 1.00 (0.89–1.13) |
| Thyroid (C73) | 11,664 | 1.00 (0.98–1.02) | 4,712 | **0.92 (0.89–0.95)** |

Abbreviations: NA: Not available due to the small number of cases, HR: Hazard ratio, CI: Confidence interval

The model was adjusted for age at screening, body mass index, age at menarche, menopausal status, age at menopause, family history of cancer, parity, breastfeeding, oral contraceptive use, smoking status, drinking status, physical activity, comorbidity status, and hormone replacement therapy use.

Bold values indicate statistically significant results.
