# Supplementary material for: Increased risk of cancer and cancer-related mortality in middle-aged Korean women with prediabetes and diabetes: a population-based study
Source: Epidemiol Health. 2023 Aug 28;45:e2023080. doi: 10.4178/epih.e2023080 (PMC10867518; doi:10.4178/epih.e2023080)
Supplement: Supplementary Material 6. — All-cause and cancer-specific mortality rates in women with normoglycemia, prediabetes, and diabetes [file epih-45-e2023080-Supplementary-6.docx]

**Supplementary Material 6.** **All-cause and cancer-specific mortality rates in women with normoglycemia, prediabetes, and diabetes**

| **Outcome** | **Normoglycemia** | | **Prediabetes** | | | **Diabetes** | | |
| --- | --- | --- | --- | --- | --- | --- | --- | --- |
|  | **No. of cases** | **Mortality rate**  **(95% CI)** | | **No. of cases** | **Mortality rate**  **(95% CI)** | | **No. of cases** | **Mortality rate**  **(95% CI)** |
| Death from any cause | 102,830 | 188.8 (187.6–190.0) | | 44,861 | 262.0 (259.6–264.4) | | 45,592 | 718.3 (711.7–724.9) |
| Death from cancer | 41,675 | 76.5 (75.8–77.3) | | 17,638 | 103.0 (101.5–104.5) | | 12,534 | 197.5 (194.0–200.9) |
| Specific cancer site |  |  | |  |  | |  |  |
| Stomach | 3,148 | 5.8 (5.6 – 6.0) | | 1,316 | 7.7 (7.3–8.1) | | 842 | 13.3 (12.4–14.2) |
| Colorectal | 4,037 | 7.4 (7.2–7.6) | | 1,751 | 10.2 (9.7–10.7) | | 1,287 | 20.3 (19.2–21.4) |
| Liver | 4,368 | 8.0 (7.8–8.3) | | 1,853 | 10.8 (10.3–11.3) | | 1,776 | 28.0 (26.7–29.3) |
| Gallbladder | 3,367 | 6.2 (6.0–6.4) | | 1,572 | 9.2 (8.7–9.6) | | 1,238 | 19.5 (18.4–20.6) |
| Pancreatic | 4,901 | 9.0 (8.7–9.3) | | 2,339 | 13.7 (13.1–14.2) | | 1,917 | 30.2 (28.9–31.6) |
| Lung | 7,316 | 13.4 (13.1–13.7) | | 3,096 | 18.1 (17.4–18.7) | | 1,963 | 30.9 (29.6–32.3) |
| Breast | 2,321 | 4.3 (4.1–4.4) | | 848 | 5.0 (4.6–5.3) | | 413 | 6.5 (5.9–7.1) |
| Cervix | 898 | 1.6 (1.5–1.8) | | 343 | 2.0 (1.8–2.2) | | 191 | 3.0 (2.6–3.4) |
| Ovary | 1,966 | 3.6 (3.5–3.8) | | 745 | 4.4 (4.0–4.7) | | 382 | 6.0 (5.4–6.6) |

Abbreviations: No.: Number of incident cases, CI: Confidence interval

Mortality rate per 100,000 person-years.
